# Centile reference curves of the ultrasound-based characteristics of the rectus femoris muscle composition in children at 4–11 years old

**DOI:** 10.3389/fped.2023.1168253

**Published:** 2023-08-10

**Authors:** Yesenia García-Alonso, Alicia M. Alonso-Martínez, Antonio García-Hermoso, Gaizka Legarra-Gorgoñon, Mikel Izquierdo, Robinson Ramírez-Vélez

**Affiliations:** ^1^ Navarrabiomed, Hospital Universitario de Navarra (HUN), Universidad Pública de Navarra (UPNA), IdiSNA, Pamplona, Spain; ^2^ CIBER of Frailty and Healthy Aging (CIBERFES), Instituto de Salud Carlos III, Madrid, Spain

**Keywords:** ultrasound, histogram, subcutaneous fat, percentile, reference values

## Abstract

Quantitative diagnostic ultrasound has been proposed as a way to characterize muscle structure, but there is a lack of normative data for children. This study aims to establish age-specific normal ranges for echo-intensity (EI), cross-sectional area (CSA), muscular thickness (MT), and subcutaneous adipose thickness (SAT) values of the rectus femoris muscle in typically developing children. The study recruited 497 children (288 boys and 209 girls) aged 4–10.9 years (mean age 7.39 years), and muscle parameters were measured using 2D B-mode ultrasound. Percentile values and reference curves were calculated using the Lambda, Mu, and Sigma method (LMS). The results showed small variation between measurements for boys compared to girls, with the most significant difference in EI, CSA, and MT values. EI decreased with age, with the most pronounced curve in boys. SAT increased in both sexes, with a slightly higher increase in girls after the age of 9.0 years. This study provides the first age-specific reference norms for the rectus femoris muscle architecture in children, and further research is needed to validate these curves and determine their clinical utility.

## 1. Introduction

Ultrasound (US) has become the preferred first-line imaging modality for children due to its noninvasive nature, low-cost, and easy accessibility (1). The concept of muscle quality has emerged as a useful construct to explore skeletal muscle function beyond age-related declines in lean body mass (1). Muscle quality and body composition are crucial factors in clinical outcomes (2). While muscle size plays a role in strength and physical functioning, physiological adaptations can occur separately in response to strength training and chronic disuse (3). However, US imaging is operator dependent, requires significant operator training, and has a limited field of view that necessitates detailed anatomical knowledge of the imaged area (4).

Different US parameters such as cross-sectional area (CSA), muscular thickness (MT), and echo-intensity (EI) can be used to quantify muscle quality. Fukumoto et al. (5) reported that MT of the knee extensor and muscle quality assessed from EI measured using this method independently contribute to isometric knee extension strength in middle-aged and elderly women (51–87 years of age). These parameters provide insight into glucose metabolism, oxidative damage, protein metabolism, intramuscular adipose tissue, capillary density, structural composition, contractility, and fatigability. Muscle quality has been significantly associated with metabolic health (5, 6), risk of cardiovascular events (7), and overall mortality (8). Multiple factors can influence muscle quality, including composition, metabolism, fat infiltration, fibrosis, and neural activation. Poor muscle strength, rather than low muscle mass, has been identified as a major determining factor for functional decline. Obesity and physical inactivity are independent risk factors for poor muscle strength (9). A higher percentage of muscle mass and better muscle quality in quadriceps, i.e., lower EI values, are strongly associated with adverse clinical outcomes (10). Therefore, understanding the factors that influence muscle quality and assessing it using US parameters can have important implications for clinical outcomes in children.

Previously, studies have evaluated the quality of skeletal muscle using computer-aided gray scale analysis, showing that EI is associated with muscle strength independently of age or muscle size in middle-aged and older adults (11). Lower EI values (12) and increased intramuscular fat (13) have been linked to impaired strength and physical function in various conditions, from injury to aging to metabolic disease (14). Lower EI values have also been associated with reduced activation of quadriceps muscles in older adults (15). Muscle mass not only affects muscle strength and mobility, but also overall survival and prognosis related to underlying diseases (15).

Given the rapidly growing population at risk in Spain (16) and the strong association between muscle US parameters and conditions such as sarcopenia or pediatric dynapenia (17), assessing muscle quality is critical for disease prevention (18). In this line, García-Alonso et al. (19) observed a relationship between physical fitness components and muscle US parameters in prepubertal children's rectus femoris muscle. With regard to subcutaneous adipose tissue (SAT) Point. Chmid-Zalaudek et al. (20) studied SAT measured via US in children and adolescents demonstrated that those with excess adiposity, as determined by DXA (%body fat), had significantly higher levels of cardio-metabolic risk factors. In this context, quantitative musculoskeletal diagnostic US has been proposed as a viable method for characterizing muscle structure (21). Reference data is necessary, as a first step, to identify individuals with low muscle quality and/or high SAT across the age spectrum. While use of the US device has become routine practice in adults, the question remains of whether both the technique and the diagnostic cutoff values for adults can be applied to youth. Moreover, published muscle quality data from healthy youth remain scarce (22). Beyond the size of a peripheral muscle, the EI of the muscle is also of great interest, since increased EI, which results from intramuscular fat and interstitial fibrous tissue, is associated with impaired physical functioning (23). Nevertheless, population-specific data are valuable in reducing the risk of misclassifying the muscle quality phenotype among children, as sociodemographic, genetic, and lifestyle factors influence body composition.

Accordingly, the purpose of the present study was to establish age-specific reference norms of EI, CSA, MT, and SAT values in children aged between four and eleven years of age without underlying metabolic disease. These reference values may assist in identifying target populations for primary prevention and guiding population health programs, policies and priorities.

## 2. Materials and methods

### 2.1. Study design and simple

Using a cross-sectional study design from the “Observatorio de Actividad Física en escolares, https://observatorioactividadfisica.es”, we examined muscle US parameters in Spanish children. The sample included 497 children aged 4.0–10.9 (288 boys and 209 girls, mean age 7.39 years). Participants were enrolled from four interested schools (a private school, Santa María la Real-Maristas; and three state schools, San Juan de la Cadena, El Lago de Mendillorri and Garcia Galdeano), two sports centers (a private sports center, S.C.D.R Anaitasuna; and a football club, Gazte Berriak C.F), and a health center (C.S Iturrama) from the Metropolitan Region of Pamplona (Navarra), Spain. This sample of the population was chosen due to the lack of muscle US parameters studies (independently of anthropometric values). Parents/guardians of children were informed of the study objectives during meetings and were invited to review the study protocol. Exclusion criteria included injury/surgery in the last month and/or any medical limitation/restrictions on physical ability testing. Informed consent was obtained from the parents/guardians and the children. Evaluations took place from December 2021 to June 2022. The study protocol was completed in accordance with the Helsinki Declaration and was approved by the Ethics Committee of the Universidad Pública de Navarra (ID # CENEDUCA1/2019).

### 2.2. Measurements

The data collection staff had a background in physical fitness and physical activity assessment and were trained by research staff from the coordinating center (e-FIT UPNA Research Group). Age and sex were assessed using a self-report questionnaire. Anthropometric measures (height, weight, and waist circumference) were collected following the CDC-NHANES survey protocol (24), by two members of the research team. Height was measured in the Frankfurt position using a stadiometer (SECA, model 213, GmbH & co. Hamburg, Germany) with 1 mm precision. Body mass was measured in light clothing and bare feet using a TANITA device scale (TANITA DC-430MAS®, Tokyo, Japan) with 100 g precision. The waist circumference measurement was taken midway between the tenth rib and the iliac crest and was recorded to the nearest millimetre. A non-elastic flexible tape measure was employed with the subject in a standing position (Seca 201, Seca GmbH & co. Hamburg, Germany). Body mass index (BMI in kg/m^2^) was subsequently derived, and BMI *z*-scores were calculated using age- and sex-specific reference data from the World Health Organization (25). The waist-to-height ratio was calculated as waist circumference/height in cm.

**Figure 1:**
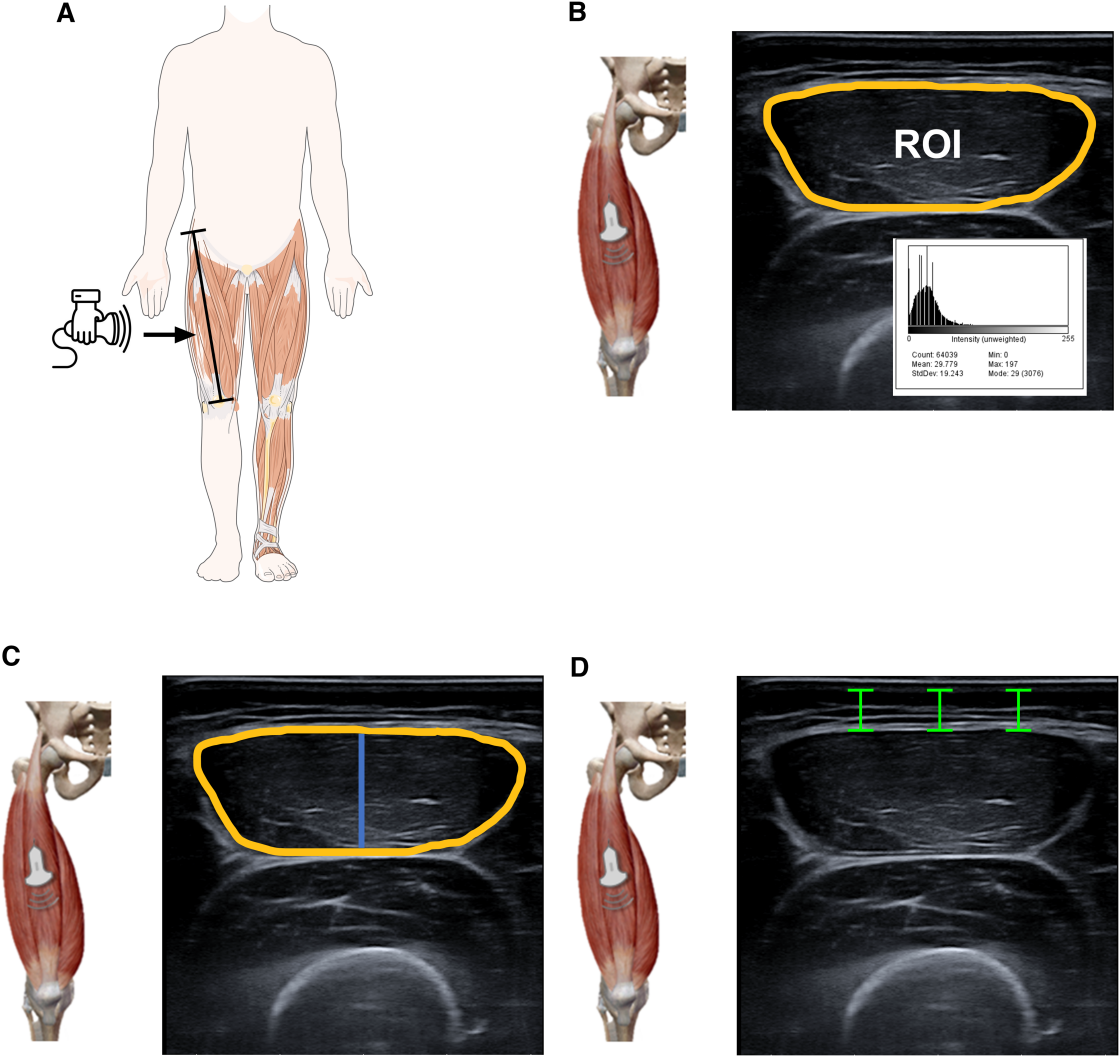
Illustrative representation of ultrasound parameters of the rectus femoris. US images were acquired at lengths equivalent to the 50% levels of the thigh length measured from the anterior superior iliac spine and the mid patella point. The transducer was positioned at the intersection points between the sagittal and transversal planes. Panel (**A**) EI was then defined as the mean level of gray within the ROI (yellow line) using the grayscale histogram function (e.g., pixels expressed as a value between 0 = black and 255 = white). Panel (**B**) The inner outline of the rectus femoris was manually traced to calculate CSA by a movable cursor on a frozen image, identified by their hyperechoic appearance. The MT (blue line) was defined as the distance between the superficial fascia and the deep fascia. Panel **(C,D**) The SAT (green line) was captured in the sagittal and transverse planes and defined as the distance between the dermis and fascia of the rectus femoris muscle using internal software on the Esaote MyLab™50. The figure shows the area of echo intensity (yellow) SAT (green) and MT (blue). SAT was quantified using the straight-line function at three sites (medial, midpoint, lateral scan) from the skin to the superficial aponeurosis and calculated as the average of the three values. The histogram displays the EI (mean) of each image. “a.u” as a unit for EI is arbitrary units.

The muscle architecture and echo intensity of rectus femoris was measured by real-time two-dimensional B-mode US (Esaote MyLab™50, Genova, Italy). Participants were asked to lie supine on a bed with extended knee joints and rest completely during the image acquisition. To ensure body fluid shift stabilization, participants were given five minutes of rest in this position. A mark was drawn at 50% of the distance between the anterior superior iliac spine and the mid patella point. The images were obtained by an expert operator (YGA) using a multi-frequency linear transducer (4–15 MHz). To ensure proper probe placement and consistent image capture location, a dotted line was drawn transversely and sagittal along the surface of the skin from the aforementioned location. All measures of muscle morphology were obtained using fixed settings that remained constant throughout the examination of each participant. This approach was employed to minimize any potential bias in the instrumentation, to optimize spatial resolution, and to ensure consistency in the measurement of muscle morphology. Image gain was set at 55 decibels (dB), dynamic range was set at 72, and image depth was set at 45–50 mm. Any optional postprocessing available within the software was switched off and time gain compensation buttons were kept in their neutral positions. Transmission gel (Ultrasound GEL® Ref: 33273, Gima s.p.A Laboratories, Inc., Gessate, MI, Italy) was used for all scans to improve acoustic contact, and minimum pressure was applied to partially visualize the muscle border. Still images were captured in both sagittal and transverse planes, followed by complete images captured with the panoramic function. Rectus femoris architecture parameters were estimated in four ways: (i) EI was determined by tracing the maximum region of interest (ROI, Figure 1B) representing the rectus femoris CSA, followed by calculating the mean level of gray within the ROI in 8-bit resolution images (gray levels from 0 to 255, where black = 0 and white = 255) using ImageJ software (ImageJ, National Institutes of Health, USA, version 1.45s). Higher scores indicated increased intramuscular fat and interstitial fibrous tissue (21). The mean and standard deviation (SD) of each histogram were computed. The inner outline of the rectus femoris was manually traced to calculate CSA by a movable cursor on a frozen image, identified by its hyperechoic appearance (Figure 1B). The MT was quantified using the line tool at the midpoint of the horizontal distance between the anterior and posterior sides of the rectus femoris. MT was measured as the minimum distance between the inferior border of the superficial aponeurosis and the superior border of the deep aponeurosis. MT value is highlighted in blue (Figure 1C) and recorded in mm. The SAT thickness was quantified using line was drawn perpendicular to the epithelium and the superior border of the superficial aponeurosis, and the resulting distance was calculated (26). Data were reported in mm, using internal software on the Esaote MyLab™50. This analysis was similar to previously established methods (21, 27), and anatomical measurement sites are described in detail in Figures 1A–C.

### 2.3. Statistical analyses

Sample were categorized according to sex into eight age categories, from 4 to 11 years. Outlier analysis was performed to verify that all values were within a physiologically possible range. Smoothed age-specific and sex- specific percentiles and curves were developed by Cole and Green (28).The least mean squares (LMS) technique estimates 3 parameters: median (M), coefficient of variation (S), and power in the Box‒Cox transformation (L). These three parameters vary as a function of independent variables, and worm plots were used to assess goodness of fit. Normality was assessed using Kolmogorov–Smirnov tests. We included in the analysis smoothed LMS curves for the 3rd, 10th, 25th, 50th, 75th, 90th and 97th percentiles of all parameters. All data are presented as the mean 95% CI. Student's *t*-test was used to determine whether significant differences were found between the descriptive characteristics using IBM SPSS version 26.0 statistical software (IBM Corporation, Armonk, NY). The level of statistical significance was set at *p* < 0.05.

## 3. Results

Table 1 shows the anthropometrics characteristics and US measurements of the participants, consisting of 288 boys and 209 girls aged 4–10.9 years, with a mean age of 7.39 (IC95% 7.22–7.56) years. BMI and waist-to-height ratio were similar in both groups (*p *> 0.05). Age, height, body mass, and waist circumference, was significantly higher in boys than in the girls’ group (*p *< 0.05). The EI and SAT (captured in the sagittal and transverse planes) were significantly greater with the girls than with the boys’ group (*p *< 0.05). We observed no significant differences in the MT or CSA between sex groups (*p *> 0.05).

**Table 1:** Anthropometrics characteristics and US measurements of the participants.

Variables	Full sample (*n *= 497)	Boys (*n *= 288)	Girls (*n *= 209)
Anthropometrics parameters			
Age (years)	7.39 (7.22;7.56)	7.64 (7.41;7.88)	7.04 (6.81;7.28)^b^
Height (cm)	124.87 (123.70;126.04)	126.63 (125.03;128.23)	122.46 (120.78;124.13)^a^
Body mass (kg)	27.24 (26.51;27.96)	28.19 (27.21;29.16)	25.94 (24.87;27.00)^a^
Body mass index (kg/m^2^)	17.08 (16.87;17.28)	17.19 (16.92;17.46)	16.93 (16.61;17.25)
Body mass index (*z*-score)	−0.00 (−0.08;0.09)	0.04 (−0.06;0.16)	−0.05 (−0.06;0.16)
Waist circumference (cm)	58.30 (57.67;58.92)	58.90 (58.08;59.73)	57.47 (56.52;58.42)^a^
Waist-to-height ratio	0.46 (0.46;0.47)	0.46 (0.46;0.47)	0.47 (0.46;0.47)
Muscle ultrasound parameters			
Transverse measurements			
EI (au)	44.82 (43.61;46.03)	43.13 (41.45;44.81)	47.15 (45.48;48.82)^a^
CSA (cm^2^)	5.36 (5.25;5.46)	5.38 (5.24;5.53)	5.32 (5.17;5.48)
MT (mm)	13.00 (12.77;13.23)	13.14 (12.83;13.48)	12.81 (12.48;13.15)
SAT (mm)	6.44 (6.17;6.72)	6.01 (5.68;6.34)	7.04 (6.59;7.49)^b^
Sagittal measurements			
SAT (mm)	6.80 (6.56;7.04)	6.36 (6.04;6.68)	7.37 (7.02;7.72)^b^

Values are means and 95% CI. Unpaired *t*-tests were utilized to compare the differences between the sex groups.

EI, echo-intensity; SAT, subcutaneous adipose tissue; MT, muscular thickness; CSA, cross-sectional area.

^a^ *P* < 0.05.

^b^ *P* < 0.01.

**Figure 2:**
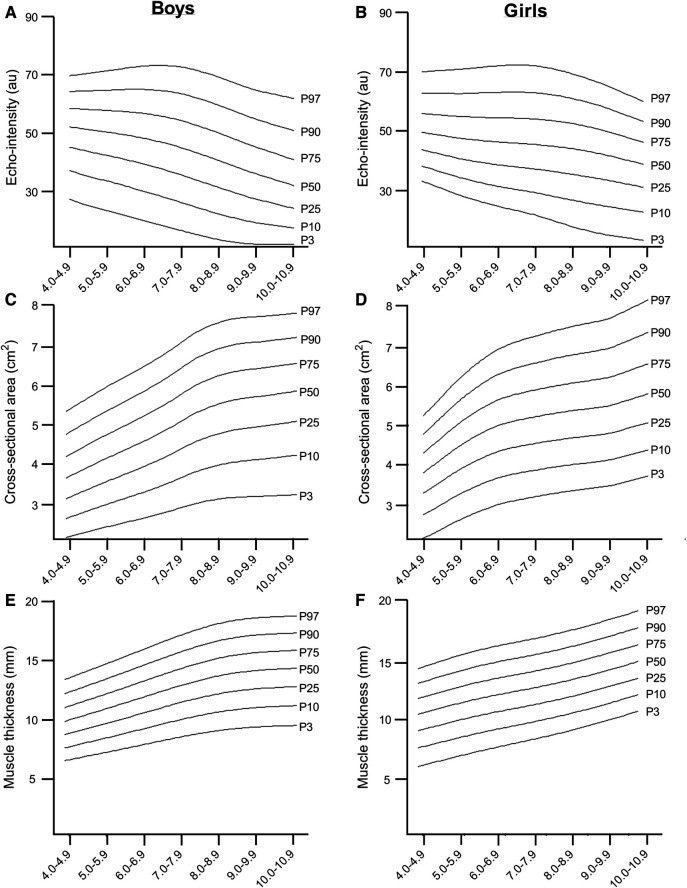
Reference curves age-specific and sex-specific of EI, CSA and MT in boys and girls.

**Figure 3:**
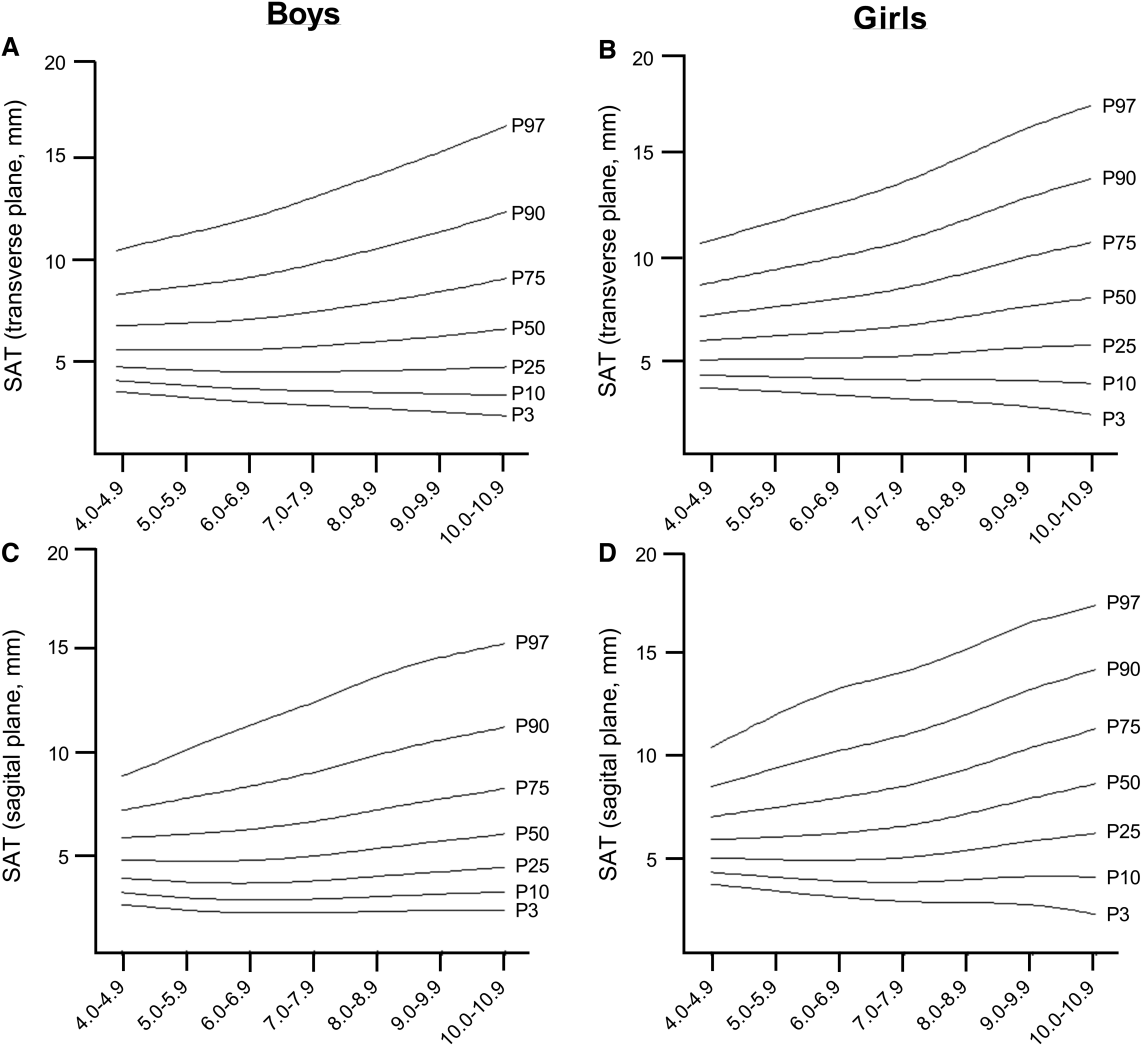
Reference curves age-specific and sex-specific of SAT in boys and girls.

The LMS reference curves, which provide information on the distribution of EI, CSA, MT, and SAT for boys and girls based on the 3rd, 10th, 25th, 50th, 75th, 90th and 97th percentiles, are summarized in Figures 2, 3, and Supplementary Tables S1–S5. We found that there were small variations between the US measurements (e.g., percentiles 90–97) and least fit (e.g., percentiles 3–10) for boys when compared to girls, particularly for EI, CSA, and MT values. For both sexes, EI decreased as age increased, with the most pronounced curve observed in boys (Figures 2A,B). The percentile curves for CSA and MT were similar for boys and girls across all ages, with differences increasing with age (Figures 2C–F). In boys, the age-related increase in CSA and MT values tends to stabilise from age 8.0 years onwards (Figures 2C–E). Similarly, SAT, as captured in the sagittal and transverse planes (Figures 3A–D), increased in both sexes, with a slightly greater increase observed in girls from age 9.0 years onwards.

## 4. Discussion

The accuracy of US in assessing skeletal muscle and its potential to predict clinical outcomes has been postulated in previous studies (29, 30). Our study highlights the use of US-based characteristics of the rectus femoris muscle composition in elementary school children, for evaluating peripheral muscle size and EI and may be helpful in monitoring muscle quality. This study should be considered as a first step, and these values can guide pediatricians who wish to apply the technique and can also be a valuable resource in the clinical assessment of muscle function and for comparisons with studies from other countries, as recommended by the European Working Group on Sarcopenia in Older People (EWGSOP2) (31), the SARCUS study (SARCopenia through UltraSound) (32), and on its possible role in diagnosing pediatric dynapenia.

US has the potential to become an imaging-based tool for screening and diagnosing skeletal muscle US parameters, comparable to computed tomography and magnetic resonance imaging, which quantify body composition on the tissue level, and dual-energy x-ray absorptiometry, which assesses the chemical level. Among qualitative measures, muscle EI provides helpful information about the presence of inflammation, fibrosis, and adipose tissue infiltration (33). Previous studies have increasingly integrated the concepts of muscular strength, peak force and body size to assess muscle performance and provide an estimate of muscle quality (34). In older adults, there appears to be a correlation between EI and muscle strength, gait speed, and sit-to-stand test (35). Among children, Garcia-Alonso et al. (19) shown that there are associations between physical fitness components and muscle US parameters in prepuberal children. In addition, muscle architectural characteristics including MT, muscle volume, EI, and CSA are strongly correlated with the maximum muscle strength and power (33, 35).

In our study, we found that boys in the 50th percentile have muscle EI values of 52.13 a.u. in the age range of 4.0–4.9 years, which decreases to 32.15 a.u. in the age range of 10.0–10.9 years. For girls, the 50th percentile, muscle EI values start at 50.32 a.u. in the age range of 4–4.9 years and decrease to 39.56 a.u. in the age range of 10.0–10.9 years. The relationship between age, CSA, and MT was found to be curvilinear, which is consistent with previous studies that have shown age-related changes in muscle mass, CSA, and muscle strength (36–38). During early childhood, there is a consistent pattern of sexual dimorphism and muscle mass quantity which is in line with previous findings from ethnically diverse populations and likely due to hormonal influences (39). We also found gender differences in SAT, which are similar to those observed in adults. In the girls group, we found an increase in SAT percentage as they age, from 5.94 mm in the 4–4.9 age group to 8.69 mm in the 10–10.9 age group (40). These gender differences seem to start from an early age and vary for upper and lower extremity muscle groups. Furthermore, we observed an age-related increase in EI mean values in children, and it is known that, in women, muscle tissue develops increased fatty infiltration, leading to increases in EI (32). Therefore, tracking the development of lean mass during adolescence can be a useful tool in identifying potential interventions for metabolic diseases associated with sarcopenia early in life (13).

Although our analyses were robust, there are several limitations that should be taken into account. Apart from sex differences, other factors, such as nutritional status, environmental factors, or ancestral differences, can influence muscle growth. To address this issue, we included only children living in the city of Pamplona (Spain) to minimalize cultural differences between ancestral groups (e.g., beliefs and traditional food) and living circumstances (e.g., housing, possibility of schools, access to sports clubs). However, it would be interesting to consider these factors in further studies or studies with older children. Additionally, our study design did not involve following up with the same group over time, so we cannot confirm if the observed trends will persist over time. Moreover, the lack of data on dietary patterns limits our ability to interpret the contribution of diet to body composition development. There is no clear criterion for muscle EI value, and the results cannot be readily generalized for different US devices, as EI is influenced by US system hardware and software. Therefore, our normative EI values can only be used with the same ultrasound device and settings, and new values must be established for other cases. However, the measurement of CSA or MT does not depend on equipment settings, so normative data of muscle size presented in this study can be applied in other centers. On the other hand, the reliability of MT, CSA and SAT measurements has been investigated in previous studies (43–45). The coefficient of variation examining the intra and inter-experimenter reliability of the US imaging technique ranged from 0.6 to 2.7%, even by minimally trained or untrained professionals. Based on these results, the experimenters concluded that US is a valid and reliable tool to assess large muscle quality parameters.

Despite these limitations, our study contributes to our understanding of how muscle parameters varies by sex and age in children. This measure can offer valuable insights regarding muscle quality of the normal rectus femoris muscle, which plays a key role in for assessing body composition in all age groups (21). Measuring muscle size and composition from magnetic resonance imaging or computed tomography is expensive and may not be accessible for youth at the population level. Since US imaging is a more accessible and less expensive technique that provides valuable information about muscle function, muscle composition testing and monitoring will provide valuable insights into the health status of youth at individual and group levels. Although there is no established cut-off for defining healthy quality muscle mass in the pediatric population, the lowest CSA and MT percentiles computed in our study (3rd and 10th percentile), or higher EI percentiles computed in our study (90th and 97th percentile), could be used as an indication of the worsening lean mass phenotype. Investigating the correlation between these muscle US parameters and early adiposity rebound could provide an effective marker of obesity in children and help tailor nutritional and exercise interventions to improve the treatment of metabolic diseases associated with sarcopenia in early life (41). Future studies combining these percentiles with functional data (e.g., muscle strength) are required for defining dynapenia and/or low-quality muscle mass among children and adolescents (13).

In situations where resources are limited and longitudinal follow-up is difficult, ultrasonographic measurements of the rectus femoris muscle can serve as a preliminary alternative. Additionally, the LMS analysis is a popular method for obtaining smoothened centile curves for cross-sectional data (42). Thus, our charts can provide valuable information to researchers studying adolescents in this geographic region and can assist healthcare providers in identifying abnormalities in body composition development during youth. In summary, we have presented age- and sex-specific reference data for normal rectus femoris muscle US parameters that are unique to children. These data allow for the identification of the risk of low-quality mass and the provision of targeted treatments, such as nutritional and exercise interventions, as well as the initiation of sports programs in schools through public policies. These measures can prevent associated outcomes, such as pediatric dynapenia, and promote healthy development in children.

## Data Availability

The raw data supporting the conclusions of this article will be made available by the authors, without undue reservation.
