# Supplementary material for: Centile reference curves of the ultrasound-based characteristics of the rectus femoris muscle composition in children at 4–11 years old
Source: Front Pediatr. 2023 Aug 10;11:1168253. doi: 10.3389/fped.2023.1168253 (PMC10449539; doi:10.3389/fped.2023.1168253)
Supplement: Supplementary file 1 [file Datasheet1.docx]

Supplementary Material

Reference Values for Muscle Ultrasound Parameters in Children

*** Correspondence:** Robinson Ramírez-Vélez, E-mail: robin640@hotmail.com

| **Table 1.** Smoothed age-specific and sex-specific percentile of echo intensity uncorrected (au) in boys and girls | | | | | | | | | | |
| --- | --- | --- | --- | --- | --- | --- | --- | --- | --- | --- |
| **Sex / age group** | **N** | **L** | **S** | **P3** | **P10** | **P25** | **P50 (M)** | **P75** | **P90** | **P97** |
| **Boys (*n*=288)** |  |  |  |  |  |  |  |  |  |  |
| 4.0−4.9 | 38 | 1.82 | 0.19 | 27.54 | 37.24 | 45.19 | 52.13 | 58.39 | 64.14 | 69.49 |
| 5.0−5.9 | 38 | 1.50 | 0.23 | 23.71 | 33.76 | 42.48 | 50.38 | 57.70 | 64.58 | 71.11 |
| 6.0−6.9 | 35 | 1.22 | 0.27 | 20.34 | 30.25 | 39.48 | 48.25 | 56.68 | 64.84 | 72.78 |
| 7.0−7.9 | 56 | 1.03 | 0.31 | 16.95 | 26.40 | 35.74 | 45.00 | 54.20 | 63.34 | 72.44 |
| 8.0−8.9 | 24 | 0.93 | 0.34 | 13.83 | 22.52 | 31.46 | 40.61 | 49.90 | 59.33 | 68.87 |
| 9.0−9.9 | 53 | 0.77 | 0.37 | 12.30 | 19.59 | 27.55 | 36.09 | 45.11 | 54.57 | 64.42 |
| 10.0−10.9 | 44 | 0.49 | 0.38 | 12.26 | 17.82 | 24.44 | 32.15 | 40.93 | 50.80 | 61.76 |
| **Girls (*n*= 208)** |  |  |  |  |  |  |  |  |  |  |
| 4.0−4.9 | 35 | 0.42 | 0.18 | 33.83 | 38.90 | 44.40 | 50.32 | 56.68 | 63.49 | 70.75 |
| 5.0−5.9 | 25 | 0.58 | 0.22 | 29.16 | 35.11 | 41.53 | 48.39 | 55.70 | 63.44 | 71.60 |
| 6.0−6.9 | 29 | 0.67 | 0.25 | 25.50 | 32.23 | 39.46 | 47.15 | 55.28 | 63.82 | 72.76 |
| 7.0−7.9 | 66 | 0.79 | 0.27 | 22.64 | 30.13 | 38.03 | 46.30 | 54.89 | 63.77 | 72.92 |
| 8.0−8.9 | 24 | 1.05 | 0.29 | 18.69 | 27.58 | 36.31 | 44.94 | 53.48 | 61.94 | 70.35 |
| 9.0−9.9 | 19 | 1.21 | 0.29 | 15.74 | 25.34 | 34.22 | 42.64 | 50.72 | 58.54 | 66.15 |
| 10.0−10.9 | 10 | 1.30 | 0.29 | 13.88 | 23.35 | 31.76 | 39.56 | 46.92 | 53.95 | 60.72 |

L, power in the Box–Cox transformation for ‘correcting’ the skewness; M, median; P, percentile; S, coefficient of variation.

| **Table 2.** Smoothed age-specific and sex-specific percentile of cross-sectional area (cm^2^) in boys and girls | | | | | | | | | | |
| --- | --- | --- | --- | --- | --- | --- | --- | --- | --- | --- |
| **Sex / age group** | **N** | **L** | **S** | **P3** | **P10** | **P25** | **P50 (M)** | **P75** | **P90** | **P97** |
| **Boys (*n*=288)** |  |  |  |  |  |  |  |  |  |  |
| 4.0−4.9 | 38 | 0.75 | 0.22 | 2.15 | 2.63 | 3.13 | 3.66 | 4.20 | 4.77 | 5.35 |
| 5.0−5.9 | 38 | 0.88 | 0.22 | 2.37 | 2.92 | 3.49 | 4.07 | 4.66 | 5.26 | 5.87 |
| 6.0−6.9 | 35 | 1.02 | 0.21 | 2.57 | 3.20 | 3.83 | 4.45 | 5.08 | 5.70 | 6.32 |
| 7.0−7.9 | 56 | 1.15 | 0.21 | 2.78 | 3.49 | 4.19 | 4.86 | 5.52 | 6.17 | 6.81 |
| 8.0−8.9 | 24 | 1.28 | 0.20 | 3.03 | 3.84 | 4.61 | 5.34 | 6.04 | 6.73 | 7.39 |
| 9.0−9.9 | 53 | 1.42 | 0.20 | 3.16 | 4.05 | 4.86 | 5.63 | 6.35 | 7.03 | 7.70 |
| 10.0−10.9 | 44 | 1.55 | 0.19 | 3.20 | 4.14 | 4.98 | 5.75 | 6.46 | 7.13 | 7.77 |
| **Girls (*n*= 208)** |  |  |  |  |  |  |  |  |  |  |
| 4.0−4.9 | 35 | 1.33 | 0.20 | 2.13 | 2.71 | 3.25 | 3.76 | 4.25 | 4.72 | 5.18 |
| 5.0−5.9 | 25 | 1.23 | 0.20 | 2.53 | 3.16 | 3.77 | 4.35 | 4.92 | 5.48 | 6.02 |
| 6.0−6.9 | 29 | 1.13 | 0.20 | 2.87 | 3.55 | 4.20 | 4.85 | 5.48 | 6.11 | 6.72 |
| 7.0−7.9 | 66 | 1.02 | 0.20 | 3.13 | 3.82 | 4.50 | 5.18 | 5.86 | 6.54 | 7.22 |
| 8.0−8.9 | 24 | 0.92 | 0.20 | 3.33 | 4.02 | 4.72 | 5.42 | 6.13 | 6.85 | 7.58 |
| 9.0−9.9 | 19 | 0.82 | 0.20 | 3.51 | 4.19 | 4.89 | 5.62 | 6.36 | 7.11 | 7.88 |
| 10.0−10.9 | 10 | 0.71 | 0.19 | 3.69 | 4.37 | 5.09 | 5.83 | 6.60 | 7.40 | 8.22 |

L. power in the Box–Cox transformation for ‘correcting’ the skewness; M. median; P. percentile; S. coefficient of variation.

| **Table 3.** Smoothed age-specific and sex-specific percentile of muscle thickness (mm) in boys and girls | | | | | | | | | | |
| --- | --- | --- | --- | --- | --- | --- | --- | --- | --- | --- |
| **Sex / age group** | **N** | **L** | **S** | **P3** | **P10** | **P25** | **P50 (M)** | **P75** | **P90** | **P97** |
| **Boys (*n*=288)** |  |  |  |  |  |  |  |  |  |  |
| 4.0−4.9 | 38 | 0.83 | 0.17 | 6.59 | 7.66 | 8.76 | 9.88 | 11.03 | 12.20 | 13.39 |
| 5.0−5.9 | 38 | 0.90 | 0.17 | 7.14 | 8.32 | 9.51 | 10.72 | 11.95 | 13.19 | 14.44 |
| 6.0−6.9 | 35 | 0.96 | 0.17 | 7.71 | 8.99 | 10.29 | 11.58 | 12.89 | 14.20 | 15.51 |
| 7.0−7.9 | 56 | 1.03 | 0.17 | 8.29 | 9.69 | 11.08 | 12.46 | 13.85 | 15.22 | 16.60 |
| 8.0−8.9 | 24 | 1.09 | 0.16 | 8.83 | 10.33 | 11.81 | 13.28 | 14.73 | 16.16 | 17.59 |
| 9.0−9.9 | 53 | 1.16 | 0.16 | 9.23 | 10.82 | 12.37 | 13.89 | 15.38 | 16.85 | 18.30 |
| 10.0−10.9 | 44 | 1.23 | 0.16 | 9.44 | 11.08 | 12.66 | 14.20 | 15.71 | 17.18 | 18.62 |
| **Girls (*n*= 208)** |  |  |  |  |  |  |  |  |  |  |
| 4.0−4.9 | 35 | 1.33 | 0.19 | 6.15 | 7.72 | 9.20 | 10.60 | 11.94 | 13.23 | 14.48 |
| 5.0−5.9 | 25 | 1.27 | 0.18 | 6.94 | 8.51 | 10.00 | 11.43 | 12.82 | 14.17 | 15.48 |
| 6.0−6.9 | 29 | 1.20 | 0.18 | 7.68 | 9.22 | 10.71 | 12.15 | 13.56 | 14.95 | 16.30 |
| 7.0−7.9 | 66 | 1.14 | 0.17 | 8.36 | 9.85 | 11.31 | 12.74 | 14.15 | 15.55 | 16.92 |
| 8.0−8.9 | 24 | 1.07 | 0.16 | 9.09 | 10.54 | 11.97 | 13.40 | 14.81 | 16.22 | 17.61 |
| 9.0−9.9 | 19 | 1.00 | 0.15 | 9.92 | 11.35 | 12.77 | 14.19 | 15.61 | 17.03 | 18.45 |
| 10.0−10.9 | 10 | 0.94 | 0.14 | 10.85 | 12.25 | 13.66 | 15.08 | 16.50 | 17.94 | 19.38 |

L. power in the Box–Cox transformation for ‘correcting’ the skewness; M. median; P. percentile; S. coefficient of variation.

| **Table 4.** Smoothed age-specific and sex-specific percentile of SAT (transverse plane, in mm) in boys and girls | | | | | | | | | | |
| --- | --- | --- | --- | --- | --- | --- | --- | --- | --- | --- |
| **Sex / age group** | **N** | **L** | **S** | **P3** | **P10** | **P25** | **P50 (M)** | **P75** | **P90** | **P97** |
| **Boys (*n*=288)** |  |  |  |  |  |  |  |  |  |  |
| 4.0−4.9 | 38 | -0.06 | 0.30 | 2.68 | 3.25 | 3.95 | 4.82 | 5.90 | 7.24 | 8.90 |
| 5.0−5.9 | 38 | -0.14 | 0.36 | 2.40 | 2.99 | 3.76 | 4.76 | 6.08 | 7.82 | 10.17 |
| 6.0−6.9 | 35 | -0.17 | 0.40 | 2.28 | 2.89 | 3.71 | 4.81 | 6.31 | 8.40 | 11.34 |
| 7.0−7.9 | 56 | -0.16 | 0.42 | 2.28 | 2.93 | 3.82 | 5.03 | 6.71 | 9.07 | 12.46 |
| 8.0−8.9 | 24 | -0.13 | 0.44 | 2.34 | 3.06 | 4.04 | 5.39 | 7.26 | 9.92 | 13.72 |
| 9.0−9.9 | 53 | -0.07 | 0.45 | 2.39 | 3.18 | 4.26 | 5.74 | 7.79 | 10.64 | 14.64 |
| 10.0−10.9 | 44 | 0.00 | 0.46 | 2.41 | 3.29 | 4.48 | 6.09 | 8.29 | 11.27 | 15.32 |
| **Girls (*n*= 208)** |  |  |  |  |  |  |  |  |  |  |
| 4.0−4.9 | 35 | -0.43 | 0.25 | 3.76 | 4.34 | 5.05 | 5.94 | 7.07 | 8.53 | 10.47 |
| 5.0−5.9 | 25 | -0.29 | 0.31 | 3.42 | 4.10 | 4.96 | 6.07 | 7.52 | 9.45 | 12.07 |
| 6.0−6.9 | 29 | -0.11 | 0.36 | 3.12 | 3.91 | 4.93 | 6.26 | 8.00 | 10.30 | 13.34 |
| 7.0−7.9 | 66 | 0.09 | 0.40 | 2.90 | 3.85 | 5.06 | 6.61 | 8.57 | 11.05 | 14.17 |
| 8.0−8.9 | 24 | 0.25 | 0.41 | 2.85 | 3.98 | 5.42 | 7.22 | 9.42 | 12.10 | 15.30 |
| 9.0−9.9 | 19 | 0.42 | 0.43 | 2.74 | 4.14 | 5.88 | 7.98 | 10.46 | 13.33 | 16.61 |
| 10.0−10.9 | 10 | 0.67 | 0.44 | 2.28 | 4.10 | 6.25 | 8.69 | 11.37 | 14.29 | 17.42 |

L, power in the Box–Cox transformation for ‘correcting’ the skewness; M, median; P, percentile; S, coefficient of variation.

| **Table 5.** Smoothed age-specific and sex-specific percentile of SAT (sagittal plane, in mm) in boys and girls | | | | | | | | | | |
| --- | --- | --- | --- | --- | --- | --- | --- | --- | --- | --- |
| **Sex / age group** | **N** | **L** | **S** | **P3** | **P10** | **P25** | **P50 (M)** | **P75** | **P90** | **P97** |
| **Boys (*n*=288)** |  |  |  |  |  |  |  |  |  |  |
| 4.0−4.9 | 38 | -0.52 | 0.27 | 3.46 | 4.00 | 4.68 | 5.56 | 6.70 | 8.25 | 10.42 |
| 5.0−5.9 | 38 | -0.43 | 0.30 | 3.24 | 3.82 | 4.56 | 5.53 | 6.82 | 8.58 | 11.08 |
| 6.0−6.9 | 35 | -0.33 | 0.33 | 3.02 | 3.64 | 4.45 | 5.51 | 6.94 | 8.92 | 11.72 |
| 7.0−7.9 | 56 | -0.24 | 0.37 | 2.85 | 3.53 | 4.42 | 5.60 | 7.21 | 9.42 | 12.54 |
| 8.0−8.9 | 24 | -0.14 | 0.40 | 2.72 | 3.47 | 4.46 | 5.79 | 7.59 | 10.06 | 13.50 |
| 9.0−9.9 | 53 | -0.05 | 0.43 | 2.58 | 3.40 | 4.51 | 5.99 | 8.01 | 10.74 | 14.46 |
| 10.0−10.9 | 44 | 0.04 | 0.46 | 2.43 | 3.34 | 4.58 | 6.25 | 8.49 | 11.49 | 15.48 |
| **Girls (*n*= 208)** |  |  |  |  |  |  |  |  |  |  |
| 4.0−4.9 | 35 | -0.33 | 0.27 | 3.59 | 4.19 | 4.94 | 5.86 | 7.04 | 8.55 | 10.54 |
| 5.0−5.9 | 25 | -0.20 | 0.30 | 3.44 | 4.13 | 4.98 | 6.06 | 7.43 | 9.20 | 11.48 |
| 6.0−6.9 | 29 | -0.07 | 0.33 | 3.26 | 4.04 | 5.03 | 6.27 | 7.84 | 9.84 | 12.39 |
| 7.0−7.9 | 66 | 0.07 | 0.37 | 3.07 | 3.97 | 5.10 | 6.53 | 8.32 | 10.57 | 13.36 |
| 8.0−8.9 | 24 | 0.20 | 0.40 | 2.92 | 3.97 | 5.31 | 6.99 | 9.07 | 11.61 | 14.69 |
| 9.0−9.9 | 19 | 0.34 | 0.43 | 2.69 | 3.94 | 5.54 | 7.51 | 9.90 | 12.74 | 16.09 |
| 10.0−10.9 | 10 | 0.04 | 0.47 | 2.73 | 4.02 | 5.95 | 7.95 | 10.27 | 12.99 | 17.88 |

L. power in the Box–Cox transformation for ‘correcting’ the skewness; M. median; P. percentile; S. coefficient of variation.

**
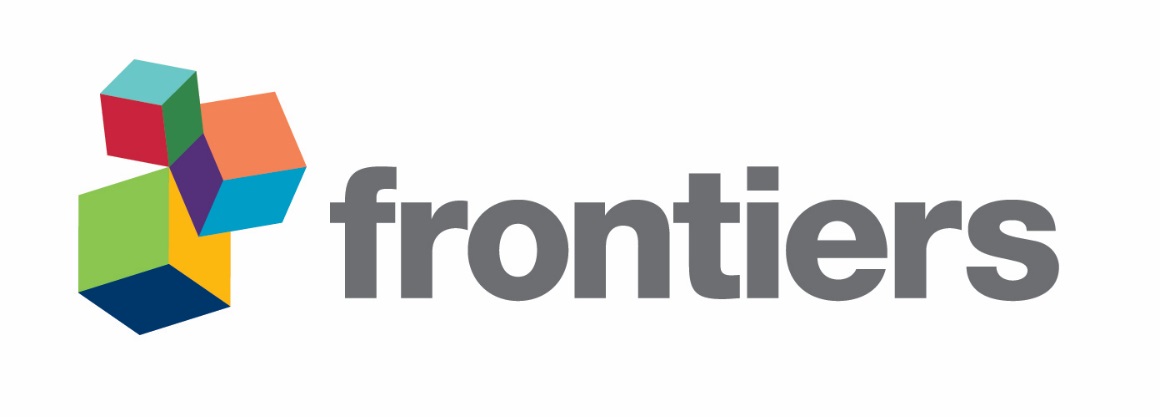
**
